# Deciphering the gut microbiota’s role in diverticular disease: insights from a Mendelian randomization study

**DOI:** 10.3389/fcimb.2024.1460504

**Published:** 2024-12-12

**Authors:** Biaohui Zheng, Dongbo Chen, Hao Zeng, Shuangming Lin

**Affiliations:** Department of Gastrointestinal Surgery, Longyan First Hospital, Longyan, China

**Keywords:** gut microbiota, instrumental variables, diverticular disease, Mendelian randomization, GWAS

## Abstract

**Background:**

Previous studies have indicated a potential association between gut microbiota and diverticular disease. However, the precise nature of this relationship remains unclear. In light of this, we decided to use a bidirectional two-sample Mendelian randomization (MR) study to investigate the causal relationship between gut microbiota and intestinal diverticular disease in greater depth.

**Methods:**

To investigate the potential causal relationship between gut microbiota and intestinal diverticular disease, we conducted a two-sample MR study in a European ancestry. Genetic instrumental variables for gut microbiota were obtained from a genome-wide association study (GWAS) involving 5,959 participants. Summary statistics for intestinal diverticular disease were sourced from the IEU Open GWAS project, which included data from 5,193 cases and 457,740 controls. The analysis was primarily conducted using the inverse variance weighted method, with additional sensitivity analyses to assess the robustness of the findings.

**Results:**

With regard to the findings, 11 microbial taxa were identified as having a potential causal relationship with intestinal diverticular disease. Specifically, the microbial taxa Caryophanales, *Paenibacillaceae*, *Herbinix*, *Turicibacter*, *Turicibacteraceae*, and *Staphylococcus fleurettii* were found to be positively associated with the risk of developing intestinal diverticular disease, while Chromatiales, *Arcobacter*, *Herbidospora*, *Ligilactobacillus ruminis*, and *Megamonas funiformis* were found to be negatively associated with the risk. Further reverse MR analysis did not reveal a reverse causal effect between these microbial taxa and intestinal diverticular disease.

**Conclusion:**

Our MR analyses revealed a potential causal relationship between certain gut microbiota and intestinal diverticular disease, which may provide new directions for future intestinal diverticular disease prevention and treatment strategies.

## 1 Introduction

Diverticular disease, a prevalent gastrointestinal disorder, is particularly significant in Western countries, with prevalence rates as high as 30% in people in their 50s and more than 70% in people older than 80 (Everhart and Ruhl, 2009). In the United States, diverticular disease results in significant healthcare utilization, with more than 1.7 million outpatient visits and over 300,000 hospitalizations annually. Additionally, it leads to approximately 38,740 30-day readmissions and 4,780 deaths, contributing to healthcare expenditures of around $9 billion annually (Peery et al., 2022).Typical symptoms of the disease include abdominal pain, diarrhea, and bleeding. Depending on the severity, diverticular disease can be subdivided into simple diverticulosis (presence of symptoms but no evidence of inflammation), diverticulitis, and diverticular hemorrhage (Tursi et al., 2020). It is important to note that diverticulitis may occur in approximately 10% to 25% of patients with diverticular disease (Strate and Morris, 2019). Therefore, studying the pathogenesis of diverticular disease (especially intestinal diverticular disease) can provide valuable references for better prevention and treatment, holding significant clinical and social value.

The gut microbiota is a complex and diverse ecosystem composed of bacteria, fungi, viruses, and other microorganisms (Lozupone et al., 2012). These microbes co-evolved with the host and play crucial roles in regulating metabolism (Hacquard et al., 2015), immunity (Hooper et al., 2012), nervous system function (Cryan and Dinan, 2012), and maintaining the intestinal barrier (Adak and Khan, 2019), earning the title of the body’s “second genome”. However, when the gut microbiota is affected by internal and external factors, an imbalance may occur, leading to reduced microbial diversity or a disrupted balance between commensal and pathogenic bacteria (Ha et al., 2014). Studies have shown that such imbalance is closely associated with various diseases, particularly gastrointestinal disorders like intestinal diverticular disease (Canakis et al., 2020; Quaglio et al., 2022; Marasco et al., 2023). For instance, research indicates that a decrease in *Clostridium* cluster IV bacteria, known for their anti-inflammatory properties, is associated with the development of intestinal diverticular disease (Barbara et al., 2017). Another prospective study found that a decline in gut microbiota diversity, coupled with a reduction in commensal bacterial families and genera (such as *Faecalibacterium* and *Ruminococcus*) and an increase in potentially pathogenic bacteria (such as *Fusobacteria*), may be linked to a higher risk of diverticulitis (Mj et al., 2022). These commensal bacteria ferment undigested dietary fiber, producing metabolites such as short-chain fatty acids (SCFAs) that not only provide energy but also protect the gut by supporting the intestinal barrier and regulating the immune system (Arumugam et al., 2011; Vinolo et al., 2011). In contrast, pathogenic bacteria release enterotoxins that disrupt tight junctions in intestinal epithelial cells, weakening the barrier function (Hecht et al., 1992). These changes collectively may damage the structure and function of the intestinal wall, thereby increasing the risk of intestinal diverticular disease (Tursi, 2016). Although a few studies (Jones et al., 2018) have suggested that there may be no direct relationship between the composition of the gut microbiota and intestinal diverticular disease, the potential impact of microbiota alterations on this condition is increasingly recognized as more research emerges. Given the lack of conclusive causal evidence, further studies remain essential.

Mendelian randomization (MR) is widely recognized for its reliability as a statistical method. The core of the method is to utilize genetic variations as instrumental variables (IVs), which in turn provide an effective assessment of the causal relationship between exposure and outcome. In comparison to traditional observational studies, MR offers significant advantages in reducing bias, avoiding confounders, and guarding against reverse causality. Currently, MR has been applied in numerous fields, and has demonstrated its unique value in exploring the causal relationship between gut microbiota and various diseases, including appendicitis, anxiety disorders, and lymphoma (Wang et al., 2023; Liang et al., 2024; Li et al., 2024).

To date, however, few studies have employed MR to explore the causal relationship between gut microbiota and diverticular disease, particularly intestinal diverticular disease. To address this gap, our study leverages summary statistics from genome-wide association studies (GWAS) and applies a bidirectional two-sample MR approach. This methodology enables us to rigorously test the potential association between gut microbiota and intestinal diverticular disease, enhancing both the stability and reliability of our findings.

## 2 Materials and methods

### 2.1 Study design

**Figure 1:**
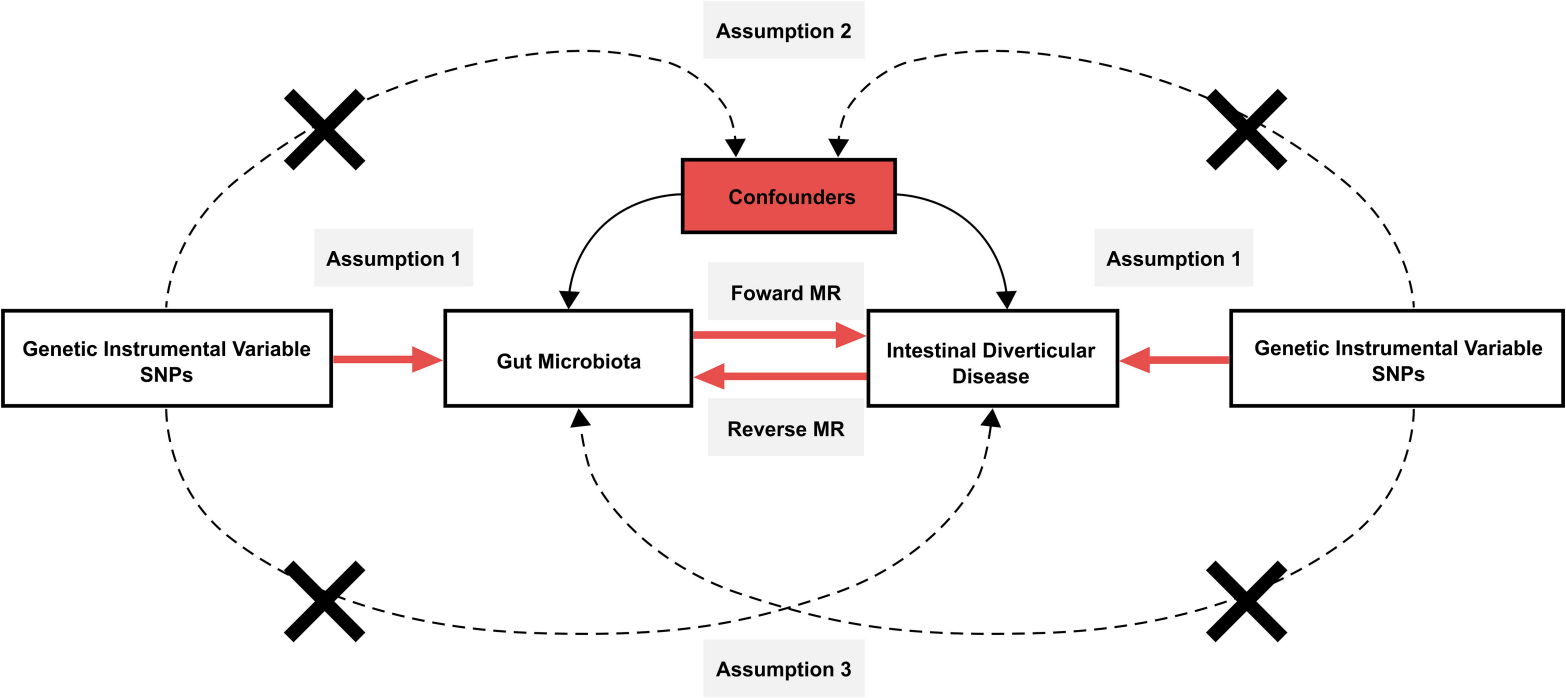
Schematic design of Mendelian randomization. Mendelian randomization requires valid genetic instrumental variables satisfying three assumptions.

In the forward MR analysis, we investigated the potential influence of gut microbiota on the development of intestinal diverticular disease. In reverse MR, we examined the effect of intestinal diverticular disease on gut microbiota. In this process, each gut microbiota and intestinal diverticular disease-associated single-nucleotide polymorphisms (SNP) serve as IVs for inferring causal effects between them. To ensure the validity of the IVs, the study had to fulfill the three core assumptions of MR (**Figure 1**). These assumptions are as follows: 1. The “correlation” assumption, where IVs are strongly associated with exposure factors; 2. The “independence” assumption, IVs are not associated with confounders; 3. The “exclusivity” assumption, where IVs are not associated with the outcome.

### 2.2 Data sources

The GWAS data on gut microbiota were obtained from the FINRISK 2002 study in Finland, a large population-based prospective cohort study (Qin et al., 2022). The study conducted a comprehensive genetic analysis of fecal samples from 5,959 participants. This entailed an exhaustive sample collection and analysis process, which covered 2,801 microbial taxa and 7,967,866 human genetic variants. Following rigorous statistical analysis and screening, the study identified 471 gut microbiota taxonomic groups, including 11 phyla, 19 classes, 24 orders, 62 families, 146 genera, and 209 species. Following further data cleansing, taxonomic units that could not be accurately identified were eliminated, and 410 units were included as the main subjects of the study.

The GWAS data for intestinal diverticular disease were obtained from the IEU Open GWAS program “ukb-b-14796” (https://gwas.mrcieu.ac.uk/). The study covered 462,933 people, of which 5,193 were intestinal diverticular disease patients and 457,740 were controls. It is worth mentioning that the population participating in the above study was of European ancestry (**Table 1**).

**Table 1:** Data sources and information used in this study.

Variable	ID	Sample size	Web resource
Gut Microbiota	PMID: 35115689	5,959	https://www.nature.com/articles/s41588-021-00991-z
Intestinal Diverticular Disease	ukb-b-14796	462,933	https://gwas.mrcieu.ac.uk/datasets/ukb-b-14796/

### 2.3 Selection of IVs

To ensure the accuracy of the causal conclusion between gut microbiota and intestinal diverticular disease, we extracted SNPs that were significantly associated with exposure as IVs. The final screened SNPs were required to satisfy the following conditions: 1) Threshold *p* < 5x10^-6^; 2) Consistent with linkage disequilibrium (LD) with R^2^ < 0.001 and LD > 10,000; 3) *F*-statistic > 10. In addition, we utilized the LDlink to exclude SNPs that may be significantly associated with potential confounders (https://ldlink.nih.gov/?tab=home).

### 2.4 Data analysis

In assessing the causal relationship between exposure and outcome, we used a variety of MR methods, including inverse variance weighted (IVW), MR-Egger, weighted median, simple mode, weighted mode, leave-one-out sensitivity analysis, and MR-PRESSO. where IVW was used as the main analytical method to derive a combined causal estimate by combining the Wald ratios of all IVs based on the assumption that all IVs are valid variables (Burgess et al., 2023). At the same time, we corrected the p-values using the Bonferroni method by setting different significance p-values at different classification levels (phylum *p*< 4.545×10^-03^, class *p*< 2.632×10^-03^, order *p*< 2.083×10^-03^, family *p*< 8.065×10^-04^, genus *p*< 3.425×10^-04^, species *p*< 2.392×10^-04^) (Sedgwick, 2014). If the p-value is between the above significance p-value and 0.05, we consider that they have a potential causal relationship. MR-Egger and MR-PRESSO can be used to detect horizontal pleiotropy (*p*< 0.05). When the intercept of MR-Egger is not zero, it may imply the existence of horizontal pleiotropy, which may violate the basic assumptions of MR analysis. Leave-one-out sensitivity analysis was used to assess the degree of dependence of the results on a single IV by removing each SNP one by one and rerunning the MR analysis to observe the stability of the results. The Q-statistics of IVW and MR-Egger were used to assess the degree of heterogeneity among IVs. The presence of heterogeneity was indicated when the p-value of the heterogeneity test was less than 0.05. All analyses were based on “TwoSampleMR”, “MRPRESSO”, “ggplot2”, “foreach” and “foreach” software packages in R version 4.3.2.

## 3 Results

### 3.1 Selection of IVs

Based on the initially set criteria, we screened 410 gut microbiota taxonomic groups and intestinal diverticular disease for suitable SNPs as IVs, and the detailed results are shown in **Supplementary Data Sheets 1–3**.

### 3.2 MR results of the effect of gut microbiota on intestinal diverticular disease

**Figure 2:**
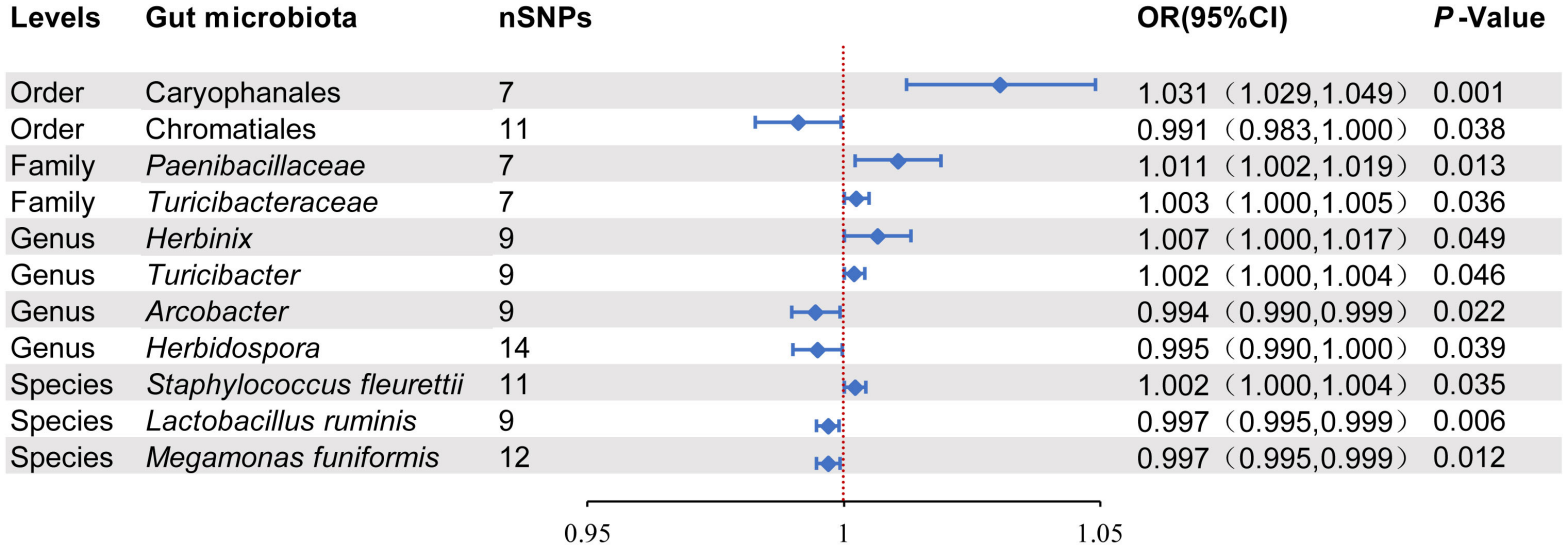
Positive MR results of causal links between gut microbiota on intestinal diverticular disease. SNP, Single-nucleotide polymorphism; OR, odds ratios; CI, Confidence interval.

**Figure 3:**
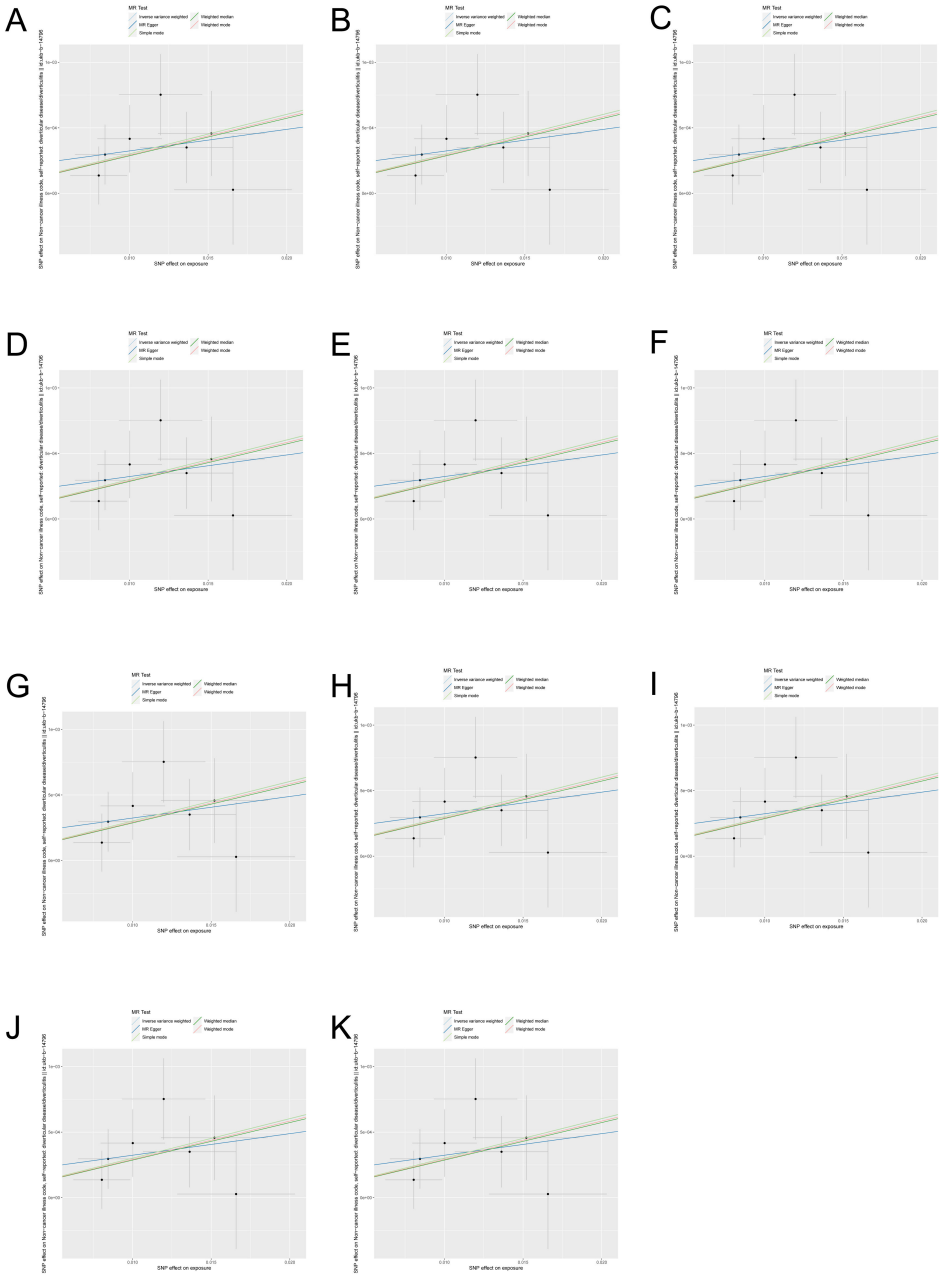
Scatter plots of gut microbiota with causal effects on intestinal diverticular disease. **(A)** Caryophanales; **(B)** Chromatiales; **(C)** *Paenibacillaceae*; **(D)** *Turicibacteraceae*; **(E)** *Herbinix*; **(F)** *Turicibacter*; **(G)** *Arcobacter*; **(H)** *Herbidospora*; **(I)** *Staphylococcus fleurettii*; **(J)** *Ligilactobacillus ruminis*; **(K)** *Megamonas funiformis*.

According to the results of the IVW analysis, there was a potential causal association between 11 microbial taxa and intestinal diverticular disease (*p* < 0.05) (**Figure 2**). Specifically, there was a positive correlation between increasing abundance of Caryophanales and diverticular disease risk at the order level (OR 1.031, 95%CI 1.029-1.049, *p*=0.001), whereas Chromatiales was negatively associated with diverticular disease risk (OR 0.991, 95%CI 0.983-1.000, and *p*=0.038). At the family level, *Paenibacillaceae* (OR 1.011, 95%CI 1.002-1.019, *p*=0.013) and *Turicibacteraceae* (OR 1.003, 95%CI 1.000-1.005, *p*=0.036) were positively associated with diverticular disease risk. At the genus level, *Herbinix* (OR 1.007, 95%CI 1.000-1.017, *p*=0.049) and *Turicibacter* (OR 1.002, 95%CI 1.000-1.004, p=0.046) were positively associated with the risk of diverticular disease, while *Arcobacter* (OR 0.994, 95%CI 0.990-0.999, *p*=0.222) and *Herbidospora* (OR 0.995, 95%CI 0.990-1.000, *p*=0.039) were negatively associated with diverticular disease risk. At the species level, *Staphylococcus fleurettii* (OR 1.002, 95%CI 1.000-1.004, *p*=0.035) was positively associated with the risk of diverticular disease, while *Ligilactobacillus ruminis* (OR 0.997, 95%CI 0.995-0.999, *p*= 0.006) and *Megamonas funiformis* (OR 0.997, 95%CI 0.995-0.999, *p*=0.012) were negatively associated with diverticular disease risk. Furthermore, the results of other MR methods are provided in the **Supplementary Table 1**, and the OR in these results are consistent with those of IVW. In order to more visually demonstrate the causal relationship between these microbial taxa and intestinal diverticular disease, we plotted a scatter plot (**Figure 3**).

### 3.3 Heterogeneity and pleiotropy of IVs

We used MR-Egger intercept and MR-PRESSO to detect pleiotropy of all IVs, and the results showed nonexistent pleiotropy (*p*>0.05). Cochran’s Q test did not find significant heterogeneity of IVs (*p*>0.05) (**Table 2**). Finally, in order to present the results of the study visually, we used leave-one-out sensitivity analysis, forest plot and funnel plot to visualize the results (**Supplementary Figures 1–3**).

**Table 2:** Heterogeneity and horizontal pleiotropy of IVs.

Exposure	Heterogeneity				Pleiotropy		
	MR-Egger		IVW		MR-Egger		MR-PRESSO
	Q	P-value	Q	P-value	Intercept	P-value	P-value
Caryophanales	3.227	0.665	3.379	0.760	1.590x10^-04^	0.713	0.802
Chromatiales	5.933	0.747	6.101	0.807	1.239x10^-04^	0.691	0.812
*Paenibacillaceae*	4.141	0.529	4.435	0.618	-2.539x10^-04^	0.611	0.649
*Turicibacteraceae*	1.976	0.853	2.185	0.902	-2.708x10^-04^	0.666	0.902
*Herbinix*	11.312	0.126	11.353	0.182	1.030x10^-04^	0.878	0.233
*Turicibacter*	3.259	0.860	3.368	0.909	-1.895x10^-04^	0.751	0.925
*Arcobacter*	6.597	0.472	6.598	0.581	-1.331x10^-05^	0.982	0.637
*Herbidospora*	10.246	0.594	10.473	0.655	-1.550x10^-04^	0.642	0.648
*Staphylococcus fleurettii*	10.721	0.295	11.510	0.319	-2.717x10^-04^	0.437	0.340
*Lactobacillus ruminis*	7.089	0.420	7.343	0.500	1.791x10^-04^	0.632	0.544
*Megamonas funiformis*	4.504	0.922	4.504	0.953	3.703x10^-06^	0.990	0.957

### 3.4 Reverse MR results

To further explore whether there was a causal effect of intestinal diverticular disease on the above 11 microbial taxa, we further performed reverse MR analysis. Finally, they were not found to be statistically associated in the IVW method (**Supplementary Table 2**).

## 4 Discussion

This study is the first of its kind to use MR to explore potential causal links between gut microbiota and intestinal diverticular disease. Based on genomic data from 5,959 individuals, we systematically analyzed the possible roles of 410 microbiota taxonomic groups in the pathogenesis of intestinal diverticular disease. Ultimately, our study revealed potential associations between changes in the abundance of 11 microbial taxa and intestinal diverticular disease. Specifically, an increased abundance of the microbial taxa Caryophanales, *Paenibacillaceae*, *Herbinix*, *Turicibacter*, *Turicibacteraceae*, and *Staphylococcus fleurettii* may promote the development of intestinal diverticular disease. In contrast, microbial taxa such as Chromatiales, *Arcobacter*, *Herbidospora*, *Ligilactobacillus ruminis*, and *Megamonas funiformis* demonstrated a potential protective effect against intestinal diverticular disease.

The gut microbiota is a vast assemblage of microorganisms that inhabit the human gut. It is comprised of hundreds of millions of microorganisms that work in concert to form a complex ecosystem (Sandler et al., 2002). They are mainly composed of the phylum Firmicutes and Bacteroidota, with a few belonging to the phylum Actinomycetota, Fusobacteria, and Pseudomonadota, among others (Eckburg et al., 2005). This microbiota composition is highly dynamic and influenced by factors such as age, diet, medication, lifestyle, and environmental exposures, which can all lead to shifts in microbial balance, often referred to as dysbiosis (Rinninella et al., 2019). Dysbiosis has been implicated in a range of systemic diseases, spanning neurological conditions like Parkinson’s disease (Wang et al., 2024), gastrointestinal disorders like inflammatory bowel disease (Lloyd-Price et al., 2019) and diverticular disease, and metabolic and immune-related diseases such as obesity (Le Chatelier et al., 2013) and autoimmune disorders (Alkader et al., 2023).

A study conducted in Italy observed a notable increase in the abundance of the phylum Firmicutes in patients diagnosed with intestinal diverticular disease, particularly within the family *Ruminococcaceae*, with levels exceeding twice those observed in the general population (Lopetuso et al., 2018). In our study, an increased abundance of five Firmicutes (Caryophanales, *Paenibacillaceae*, *Herbinix*, *Turicibacter*, and *Staphylococcus fleurettii*) was associated with an increased risk of intestinal diverticular disease, whereas two Pseudomonadota (Chromatiales and *Arcobacter*) demonstrated a protective effect against intestinal diverticular disease. Further, an analysis of fecal samples from 28 patients with diverticulosis revealed a link between diverticulitis and increased abundance of *Pseudobutyrivibrio*, *Bifidobacterium*, and *Christensenellaceae* (Kvasnovsky et al., 2018). This supports the observed pathogenic role of *Herbinix* in intestinal diverticular disease, given its familial association with *Pseudobutyrivibrio* (both belonging to *Lachnospiraceae*). Although the odds ratios of these microbiota are not particularly large, it is important to note that their cumulative effect, when acting synergistically, could still have a meaningful impact on the development of intestinal diverticular disease. Furthermore, the majority of microbiota positively associated with intestinal diverticular disease in this study belonged to the phylum Firmicutes, which aligns with previous studies linking Firmicutes bacteria to this disease. This consistency further reinforces the reliability of our findings.

Another descriptive, cross-sectional study showed a trend toward a decrease in the number of *Clostridium* cluster IX and *Lactobacillaceae* in symptomatic intestinal diverticular disease patients, which is consistent with the protective effect of *Ligilactobacillus ruminis* against intestinal diverticular disease found in our study (Barbara et al., 2017). This mechanism may be related to the ability of these bacteria to produce SCFAs (including acetic, propionic, and butyric acids). SCFAs play a multifaceted role in supporting intestinal health. They not only activate anti-inflammatory factors, such as IL-10, which help modulate immune responses, but also stimulate B cells to produce immunoglobulin A (IgA) (Hecht et al., 1992). This production of IgA is essential for reinforcing the gut’s immune defense, as IgA binds to pathogens and toxins, preventing them from penetrating the intestinal lining. In line with this, *Megamonas funiformis*, identified in our study as a protective bacterial group, demonstrates similar beneficial functions. Originally isolated from healthy human feces, *Megamonas funiformis* has recently been shown to alleviate fatty liver disease associated with metabolic dysfunction through its production of propionic acid (Sakon et al., 2008; Yang et al., 2023). This finding suggests it may exert comparable protective effects in intestinal diverticular disease by enhancing gut health and resilience. These insights reinforce the potential application of specific “probiotics” in treating or managing intestinal diverticular disease.

*Staphylococcus fleurettii* belongs to the genus *Staphylococcus*. Although there is a relative paucity of studies on its specific association with intestinal diverticular disease, *Staphylococcus* are widely recognized to be strongly associated with a variety of infections involving multiple sites such as the intestinal tract, urinary tract, and skin (Trautner and Darouiche, 2004; Raineri et al., 2022; Severn and Horswill, 2023). In addition, it is also worth noting that *Staphylococcus fleurettii* was initially isolated from goat cheese, a finding that further corroborates speculation in previous studies about a potential link between dietary patterns and intestinal diverticular disease (Lemes et al., 2021). *Herbidospora* has been isolated primarily from soil and plant samples. Although studies on its functionality in the gut are still insufficient, scientific studies have found that its subspecies, such as *Herbidospora daliensis*, have significant anti-inflammatory properties (Kudo et al., 1993; Chen et al., 2022). Based on this finding, it is reasonable to hypothesize that *Herbidospora* may have a protective effect against intestinal diverticular disease.

This study has significant advantages. First, this study is based on MR analysis of large-scale GWAS data, which effectively overcomes the limitations of insufficient sample size, confounding factor interference and reverse causation in observational studies. Second, by selecting a study population of European origin, this study effectively reduces the influence of ethnic differences on the study results.

However, there are several limitations to this study. First, the research focuses exclusively on a European cohort, which may limit the generalizability of the findings to other populations. Additionally, we acknowledge that the pathogenic mechanisms of intestinal diverticular disease may vary depending on its location and type. Due to data limitations, our study was unable to further differentiate between specific locations or types of intestinal diverticular disease. However, the majority of the data are based on patients with colonic intestinal diverticular disease, meaning that the results primarily reflect the relationship between gut microbiota and colonic intestinal diverticular disease. Finally, the lack of detailed information on age and lifestyle factors restricted further stratified analyses. Future research should aim to validate these findings, address these limitations, and explore in more detail the biological mechanisms underlying the relationship between gut microbiota and intestinal diverticular disease.

## 5 Conclusion

This study, employing a bidirectional two-sample MR approach, identifies potential causal relationships between 11 gut microbiota taxa and intestinal diverticular disease. The findings suggest that variations in microbiota abundance may influence the onset and progression of intestinal diverticular disease, with some taxa providing a protective effect and others increasing risk. Future research should validate these results across diverse ethnic groups and regions (such as Asia and Africa) to assess the impact of racial and geographic differences on the gut microbiota–disease relationship. Integrating functional genomics and experimental studies will be essential to further investigate the role of symbiotic and pathogenic bacteria in the pathogenesis of intestinal diverticular disease and gut ecological balance. Additionally, considering lifestyle factors, dietary habits, and their interactions with microbiota will be crucial in understanding their influence on disease risk. The effect of gut microbiota on intestinal diverticular disease in various sites and forms should also be explored.

In summary, this study expands our understanding of the gut microbiota–intestinal diverticular disease relationship, providing valuable insights for the development of personalized treatment and prevention strategies.

## Data Availability

The datasets presented in this study can be found in online repositories. The names of the repository/repositories and accession number(s) can be found in the article/**Supplementary Material**.
