# Supplementary material for: Deciphering the gut microbiota’s role in diverticular disease: insights from a Mendelian randomization study
Source: Front Cell Infect Microbiol. 2024 Dec 12;14:1460504. doi: 10.3389/fcimb.2024.1460504 (PMC11669361; doi:10.3389/fcimb.2024.1460504)
Supplement: Supplementary file 4 [file Image1.pdf]

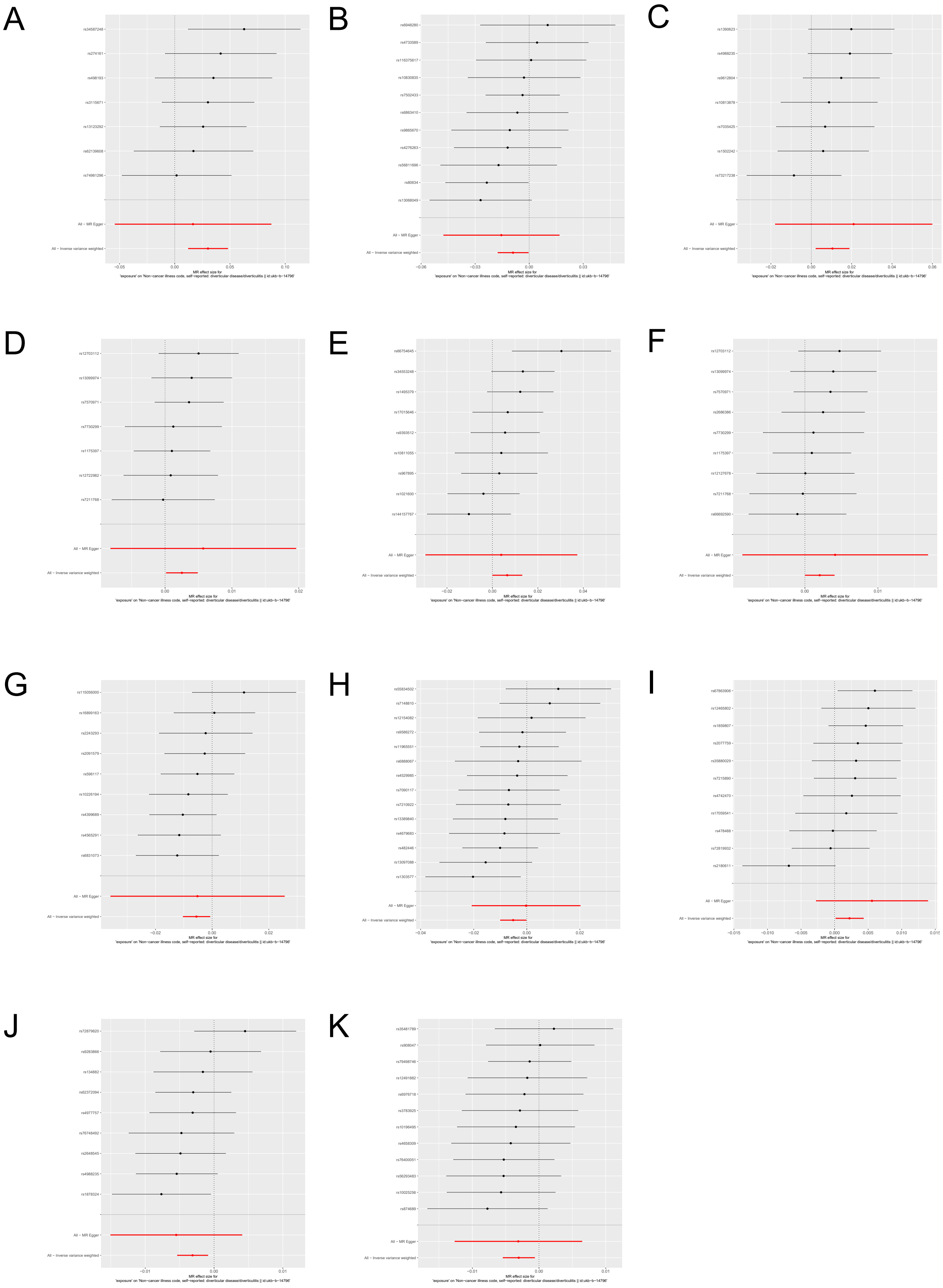

Supplementary Figure 1. Forest plots of gut microbiota with causal effects on intestinal diverticular disease. (A) Caryophanales; (B) Chromatiales; (C) *Paenibacillaceae*; (D) *Turicibacteraceae*; (E) *Herbinix*; (F) *Turicibacter*; (G) *Arcobacter*; (H) *Herbidospora*; (I) *Staphylococcus fleurettii*; (J) *Ligilactobacillus ruminis*; (K) *Megamonas funiformis*.

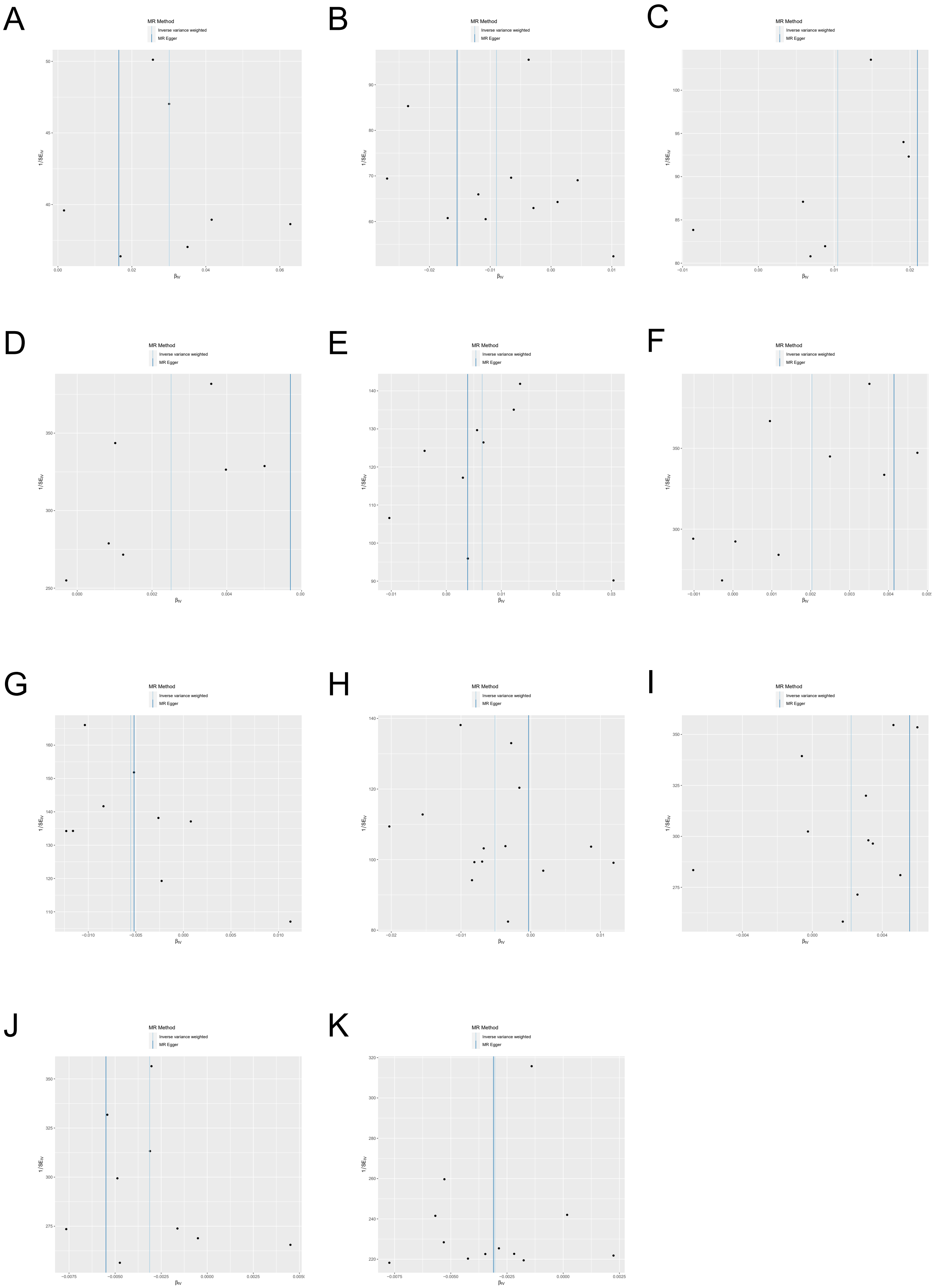

Supplementary Figure 2. Funnel plots of gut microbiota with causal effects on intestinal diverticular disease. (A) Caryophanales; (B) Chromatiales; (C) *Paenibacillaceae*; (D) *Turicibacteraceae*; (E) *Herbinix*; (F) *Turicibacter*; (G) *Arcobacter*; (H) *Herbidospora*; (I) *Staphylococcus fleurettii*; (J) *Ligilactobacillus ruminis*; (K) *Megamonas funiformis*.

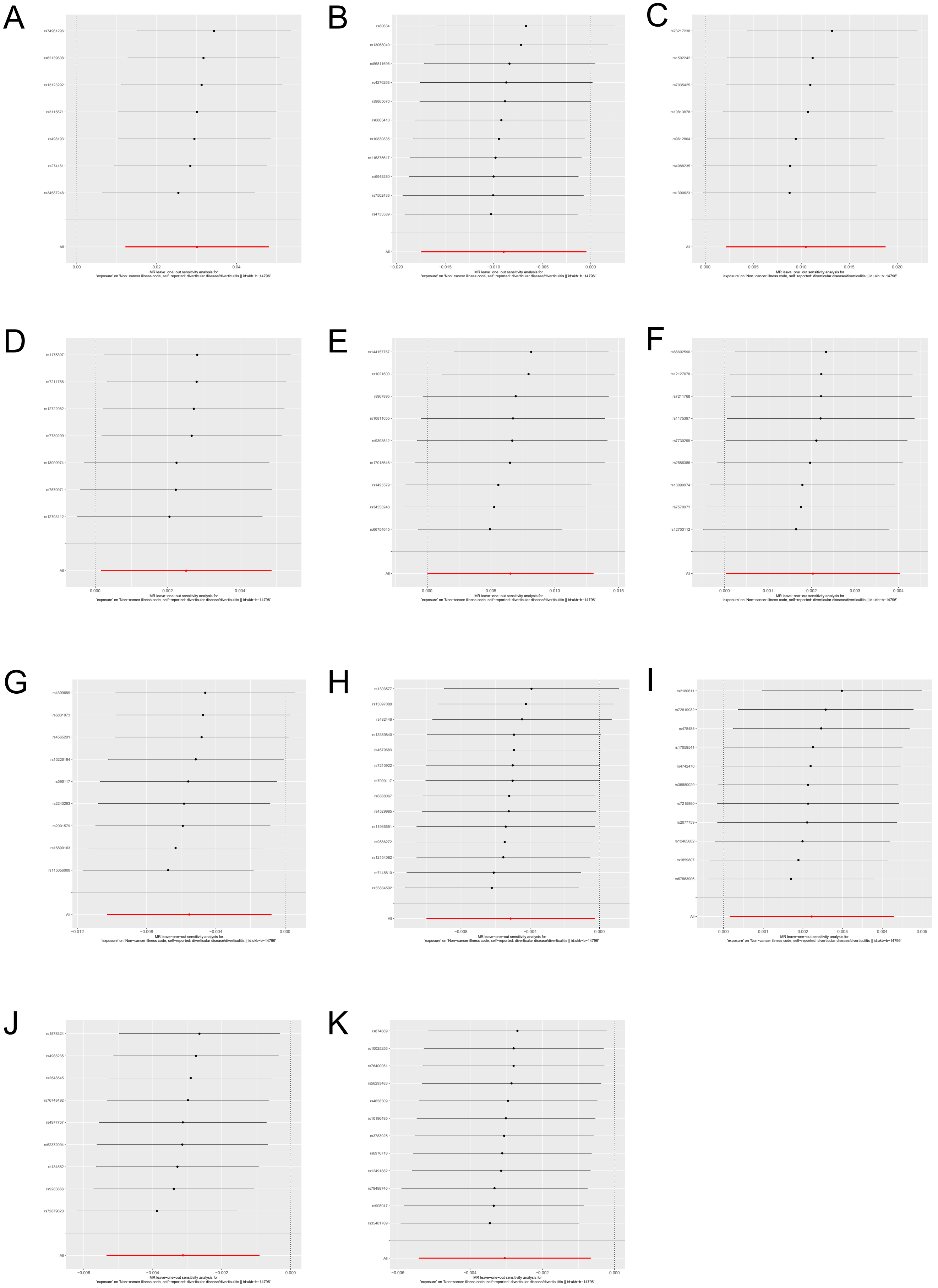

Supplementary Figure 3. Leave-one-out sensitivity analysis of gut microbiota with causal effects on intestinal diverticular disease. (A) Caryophanales; (B) Chromatiales; (C) *Paenibacillaceae*; (D) *Turicibacteraceae*; (E) *Herbinix*; (F) *Turicibacter*; (G)*Arcobacter*; (H) *Herbidospora*; (I) *Staphylococcus fleurettii*; (J) *Ligilactobacillus ruminis*; (K) *Megamonas funiformis*.
